# Conserved Roles of CrRLK1L Receptor-Like Kinases in Cell Expansion and Reproduction from Algae to Angiosperms

**DOI:** 10.3389/fpls.2016.01269

**Published:** 2016-08-29

**Authors:** Sergio Galindo-Trigo, Julie E. Gray, Lisa M. Smith

**Affiliations:** ^1^Department of Animal and Plant Sciences, University of SheffieldSheffield, UK; ^2^Department of Molecular Biology and Biotechnology, University of SheffieldSheffield, UK

**Keywords:** CrRLK1L, signaling pathway, cell expansion, functional conservation, kinase, Streptophyta

## Abstract

Receptor-like kinases (RLKs) are regulators of plant development through allowing cells to sense their extracellular environment. They facilitate detection of local endogenous signals, in addition to external biotic and abiotic stimuli. The *Catharanthus roseus* RLK1-like (CrRLK1L) protein kinase subfamily, which contains FERONIA, plays a central role in regulating fertilization and in cell expansion mechanisms such as cell elongation and tip growth, as well as having indirect links to plant–pathogen interactions. Several components of CrRLK1L signaling pathways have been identified, including an extracellular ligand, coreceptors, and downstream signaling elements. The presence and abundance of the CrRLK1L proteins in the plant kingdom suggest an origin within the Streptophyta lineage, with a notable increase in prevalence in the seeded land plants. Given the function of the sole CrRLK1L protein in a charophycean alga, the possibility of a conserved role in detection and/or regulation of cell wall integrity throughout the Strephtophytes is discussed. Orthologs of signaling pathway components are also present in extant representatives of non-vascular land plants and early vascular land plants including the liverwort *Marchantia polymorpha*, the moss *Physcomitrella patens* and the lycophyte *Selaginella moellendorffii*. Deciphering the roles in development of the CrRLK1L protein kinases in early diverging land plants will provide insights into their ancestral function, furthering our understanding of this diversified subfamily of receptors in higher plants.

## Receptor-Like Kinases (RLKs) in Plants

Plant cells sense their extracellular environment and moderate their developmental programs accordingly. Extracellular signals range from tissue-specific cues to indicators of abiotic and biotic environmental conditions such as drought or disease. A critical component of environment-sensing is plasma membrane-localized receptors. Receptor-like kinases (RLKs) are among the most expanded protein families in plants, comprising ∼600 members in *Arabidopsis thaliana* (Shiu and Bleecker, 2001). A small proportion of plant RLKs have been characterized, with comprehensive functional descriptions for relatively few members, including BRASSINOSTEROID-INSENSITIVE 1, CLAVATA1 and FLAG-ELLIN-SENSITIVE 2 (Wang et al., 2001; Chinchilla et al., 2006; DeYoung et al., 2006). Understanding RLKs is critical as they regulate many aspects of plant function from development to stress responses.

Receptor-like kinases have a modular organization consisting of an amino-terminal extracellular domain (ECD), a transmembrane (TM) domain, and an intracellular kinase domain (Walker, 1994). Classification by their variable ECDs defines 15 RLK subfamilies including the leucine-rich repeat (LRR) and *Catharanthus roseus* RLK 1-like (CrRLK1L) subfamilies (Shiu and Bleecker, 2003). The RLKs share a common mechanism for signal perception and transmission. Firstly, the ECD recognizes a specific ligand, the biochemical nature of which depends on the ECD, with ligand binding inducing receptor-coreceptor dimerization. This intermolecular interaction promotes signal transduction through conformational change, leading to kinase domain activation via auto- or trans-phosphorylation (Afzal et al., 2008). Finally, phosphorylation of downstream pathway components results in signal transmission and activation of adaptive responses to the extracellular stimulus.

## The *Catharanthus roseus* RLK1-Like (CrRLK1L) Subfamily

Named after *Catharanthus roseus*, the species in which its first member (CrRLK1) was identified (Schulze-Muth et al., 1996), the CrRLK1L subfamily has received increasing attention over the past decade. Members of the CrRLK1L subfamily have two ECD regions with similarity to the putative carbohydrate-binding malectin domain (MD). Malectin, first described in Schallus et al. (2008) in *Xenopus laevis*, acts in the endoplasmic reticulum *N*-glycosylation surveillance system.

**FIGURE 1:**
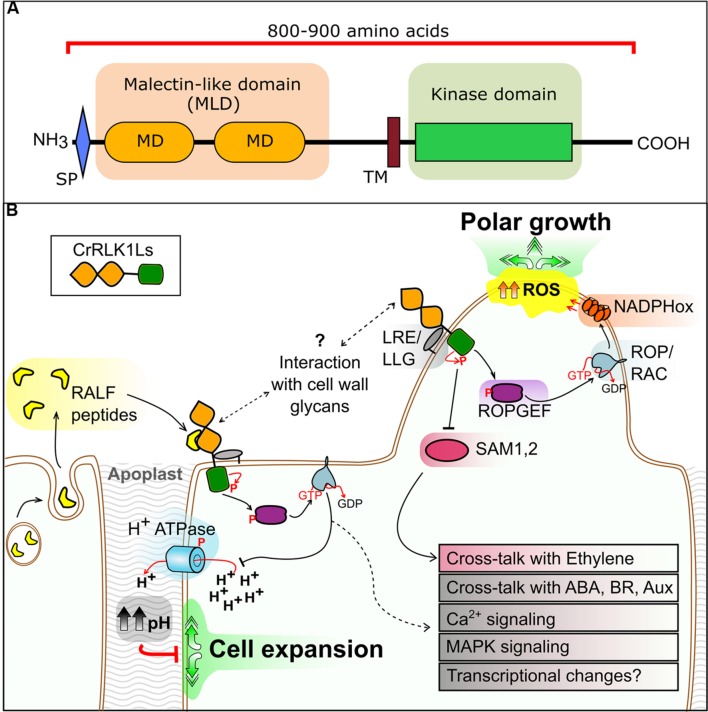
**(A)** Domain organization of the CrRLK1L proteins. Domains are to scale and based on FER as given in the NCBI Conserved Domain Database. SP, signal peptide; MD, malectin domain; TM, transmembrane domain. **(B)** Overview of CrRLK1L protein kinase signaling pathway components as described in the main text. RALF, rapid alkalinization factor; LRE/LLG, LORELEI/LRE-like GPI-AP; ROPGEF, RAC/ROP guanine exchange factor; ROP/RAC, Rho of plants/RAC GTPase; SAM1, 2, *S*-adenosylmethionine synthetase 1, 2; NADPHox, NADPH oxidase; ROS, reactive oxygen species. Speculative steps are represented by dashed lines.

The two tandem MDs are annotated as a malectin-like domain (MLD) (**Figure 1A**), a conformation specific to algae and plants. Within the RLK family, the MLD comprises the main ECD in the CrRLK1L, LRK10L-2, LRR-1c, and LRR-1a subfamilies. The MLD is classified as a rapidly evolving domain in the CrRLK1L subfamily (Escobar-Restrepo et al., 2007). Although, MLDs are highly divergent in primary sequence, suggesting functional divergence, conservation of predicted secondary structures allows inference of a role in carbohydrate binding (Boisson-Dernier et al., 2011). However, while the structure of malectin is available (Schallus et al., 2008, 2010), no three-dimensional structure of the MLD has been resolved, preventing a more definitive comparison.

## Cellular Functions of CrRLK1L Receptor-Like Kinases

Since Escobar-Restrepo et al. (2007) discovered a role for FERONIA (FER, AT3G51550) in pollen tube (PT) reception at the female gametophyte, the CrRLK1L subfamily has been linked to an increasing number of biological processes. Generally regarded as cell wall stability sensors, CrRLK1L proteins have been implicated in cell elongation and cell shape, polarized growth, and plant–pathogen interactions (reviewed in Boisson-Dernier et al., 2011; Cheung and Wu, 2011; Lindner et al., 2012; with a summarizing table of CrRLK1L functions in Nissen et al., 2016).

### Cell Wall Integrity

Genetics provided the first evidence of CrRLK1L surveillance of cell wall stability (Hematy et al., 2007), with mutations in *THESEUS1* (*THE1*, *AT5G54380*) partially rescuing the hypocotyl growth defect of cellulose deficient *cesA6* (*cellulose synthase A, catalytic subunit 6*) mutants without influencing the cellulose deficiency (Hematy et al., 2007). THE1 activation may be cell wall perturbation-dependent, leading to reinforcement by ectopic lignification, and preventing excessive loosening and consequent damage to the cell wall.

### Cell Elongation

Cell elongation during vegetative growth involves several CrRLK1L subfamily members. *HERCULES RECEPTOR KINASES 1* and *2* (*HERK1* and *HERK2*, *AT3G46290* and *AT1G30570*), *THE1*, *FER*, *CURVY1* (*CVY1*, *AT2G39360*) and uncharacterized CrRLK1L *AT5G24010* are among the most up-regulated genes upon brassinosteroid (BR) treatment (Guo et al., 2009a,b). BR functions in cell elongation and Guo et al. (2009a,b) described reduced hypocotyl and petiole cell elongation in *herk1the1* and *fer* mutants, and a stronger phenotype in the *herk1herk2the1* triple mutant. BR treatment further reduces hypocotyl length in these lines, indicating redundancy between cell elongation pathways (Guo et al., 2009a).

Balancing of cell wall rigidification/loosening has been proposed as the underlying regulatory mechanism for CrRLK1L-mediated cell elongation (Hematy et al., 2007). Cell wall-loosening enzymes, such as expansin and pectin lyase-like genes, are down-regulated in the *herk1the1* double mutant, which could promote cell wall stiffening and reduced cell elongation (Guo et al., 2009a; Boisson-Dernier et al., 2011). In contrast, expansin and pectin lyase-like gene expression is up-regulated in *the1* mutants in a *cesA6* background, suggesting context-dependent function.

### Tip Growth in Root Hairs, Pollen Tubes, and Trichomes

Tip growth represents an extreme form of polarized cell expansion that occurs during development of root hairs, PTs, and trichomes (Yang, 1998). For tip growth to occur, various mechanisms must be spatiotemporally coordinated, including polarized exocytosis of vesicles, cytoskeletal reorganization, and generation of second messenger gradients (Lee and Yang, 2008). Many key genetic and exogenous factors have been described (Li et al., 1999; Baluska et al., 2000; Rigas et al., 2001; Foreman et al., 2003; Bloch et al., 2011). Genetic factors include five CrRLK1L proteins: FER, ANXUR1 (ANX1, AT3G04690), ANX2 (AT5G28680), CVY1 and [Ca^2+^]_cyt_-associated protein kinase 1/ERULUS (CAP1/ERU, AT5G61350). Although, described in different structures (FER and CVY1 in root hairs; ANX1 and ANX2 in PTs), the former four proteins are localized to the plasma membrane of the growing tip where they increase reactive oxygen species (ROS) production by a common mechanism (Duan et al., 2010; Gachomo et al., 2014). Both *fer* and *cvy1* mutants have defects in other tip growth-requiring structures such as the leaf trichomes (Duan et al., 2010; Gachomo et al., 2014). In contrast, CAP1 is localized to the tonoplast in root hairs where it activates nitrogen permease in response to ammonium/nitrogen levels and facilitates accumulation of Ca^2+^ (Bai et al., 2014), demonstrating the potential for CrRLK1L proteins to function in a wider range of cellular processes.

### Pollen Tube-Female Gametophyte Interactions

During fertilization in angiosperms, the PT grows through the pistil, transporting two sperm cells. At the female gametophyte synergid cells (SCs), the PT bursts to release the sperm cells (Dresselhaus and Franklin-Tong, 2013). Three CrRLK1L subfamily members regulate this process in *Arabidopsis*: FER, ANX1, and ANX2. Maternal *fer* mutants fail to induce PT rupture and growth arrest (Escobar-Restrepo et al., 2007). FER is asymmetrically localized to the filiform apparatus in the SCs, and regulates a local increase in ROS (Escobar-Restrepo et al., 2007). Exogenous quenching of ROS in the SC results in a *fer*-like PT overgrowth phenotype, implying FER-triggered, ROS-mediated, PT growth arrest (Duan et al., 2014). Domain swaps between FER and closely related CrRLK1Ls indicate that, while the FER ECD is needed to complement reproductive PT reception defects in *fer* mutants, the kinase domain can be replaced with that of related CrRLK1Ls (Kessler et al., 2015). Furthermore, FER kinase activity is not required, suggesting that co-receptors may compensate or provide signal transduction capacity (Kessler et al., 2015).

The two closest homologs to FER, ANX1 and ANX2, act redundantly as its male equivalents. While *FER* is expressed in all tissues except for mature pollen, ANX1 and ANX2 localization is restricted to the PT tip (Boisson-Dernier et al., 2009). Both ANX proteins are proposed to sense cell wall integrity and maintain its stability at the PT tip (Boisson-Dernier et al., 2009, 2011; Miyazaki et al., 2009). ROS production and a Ca^2+^ gradient are required downstream of ANX1 and ANX2 in polarized PT growth (Boisson-Dernier et al., 2013).

### Biotic and Abiotic Responses

The CrRLK1L subfamily is linked to plant–pathogen interactions, with multiple CrRLK1L genes up-regulated upon elicitor treatments including bacterial flagellin (epitope flg22) and fungal chitin (Lindner et al., 2012). FER becomes phosphorylated upon treatment with flg22 and is speculated to act as a coreceptor with FLS2 (Keinath et al., 2010). FER triggers an intracellular ROS burst in *Arabidopsis* suspension cells in response to flg22 (Keinath et al., 2010). Kessler et al. (2010) identified FER as a pathogen resistance determinant in powdery mildew infections. They described the similarity between PT reception at the SCs and the first steps in infection, identifying shared molecular components (Kessler et al., 2010). In addition, a member of the mildew resistance locus O (MLO) family, NORTIA (NTA, AT2G17430), re-locates to the filiform apparatus upon PT reception at the SCs in a FER-dependent fashion and is necessary for correct PT response (Kessler et al., 2010). Together these results shed new light on plant–pathogen interactions.

Additionally, CrRLK1L genes respond to a variety of stress and hormone treatments. From genome-wide expression data, Lindner et al. (2012) summarized the general down-regulation of CrRLK1L subfamily expression in response to abiotic stresses such as heat, drought, high osmolarity, cold and hypoxia. Since CrRLK1Ls are linked to ROS production, down-regulation could indicate a strategy to minimize the deleterious oxidative burst that is common to abiotic stress responses. Hormone treatments such as BRs also increase transcript levels of a subset of CrRLK1Ls (Guo et al., 2009a,b).

## CrRLK1L Signaling Pathways

A number of proteins have been identified as either direct interaction partners of CrRLK1L kinases, or as phosphorylated/activated upon CrRLK1L signaling. Although our understanding is incomplete, conserved signaling mechanisms are emerging. CrRLK1L signaling generally involves activation of downstream elements through phosphorylation of guanine exchange factors, activation of plasma membrane NADPH oxidase-dependent ROS production and calcium ion fluctuations (**Figure 1B**).

### Upstream Effectors

Perhaps the most striking recent discovery is identification of a ligand for FER. A phosphoproteomic screen of seedlings exposed to the peptide rapid alkalinization factor 1 (RALF1, AT1G02900) identified FER as its receptor (Haruta et al., 2014). Additionally, putative downstream elements of RALF1-FER intracellular signaling were identified, including a plasma membrane H^+^-ATPase that increases apoplastic pH and cell wall rigidity upon activation (**Figure 1B**).

Surprisingly, as MDs were anticipated to bind carbohydrate ligands, RALF1 is a small secreted peptide that lacks *N*-glycosylation (Haruta et al., 2014). The RALF family comprises 34 members in *Arabidopsis*. The ubiquitous, pollen-specific or SC-specific expression patterns of some RALF-encoding genes correlate with those of CrRLK1L genes, leading to the hypothesis that additional RALF peptides may be ligands for CrRLK1Ls in different processes or developmental stages (Murphy and De Smet, 2014; Wolf and Hofte, 2014). For example, two RALF peptides, SlRALF in tomato and AtRALF4 in *Arabidopsis*, regulate PT elongation (Covey et al., 2010; Morato do Canto et al., 2014). Further evidence supporting a link between the CrRLK1L and RALF families was provided when Srivastava et al. (2009) described down-regulation of AtRALF23 upon BR treatment. AtRALF23 appears to counteract BR effects on cell growth and elongation (Srivastava et al., 2009), a process in which HERK1, HERK2, FER and THE1 participate (Guo et al., 2009a,b). Additionally, RALF1 suppresses BR effects on root cell elongation by inducing several BR-down-regulated genes involved in BR biosynthesis (Bergonci et al., 2014).

Although unexpected, the discovery of a peptide ligand for FER does not exclude carbohydrate binding by CrRLK1L proteins. As suggested by Wolf and Hofte (2014), the RALF1-CrRLK1L interaction may not involve the MLD, and each CrRLK1L kinase may have multiple biochemically diverse ligands, a property described for animal receptors such as the epidermal growth factor receptor and beta-adrenergic receptor kinase (Touhara, 1997; Moghal and Sternberg, 1999).

### Coreceptors and Chaperones

Other RLKs such as FLS2 and CrRLK1L subfamily member HERK1 may act as coreceptors with FER (Guo et al., 2009a; Keinath et al., 2010), however, only two glycosylphosphatidylinositol-anchored proteins (GPI-APs) have been biochemically confirmed to interact with FER and mediate downstream signaling (Li et al., 2015; Liu et al., 2016). LORELEI (LRE) and LRE-like GPI-AP1 (LLG1) bind the extracellular juxtamembrane domain of FER and, in different developmental contexts, are essential for deposition and stability of FER as well as effective RALF1-FER signaling (Li et al., 2015). Considering that there are two further LRE homologs in *Arabidopsis* (LLG2 and LLG3) with different expression patterns (Capron et al., 2008; Tsukamoto et al., 2010), we speculate that different LLGs could act as chaperones and co-receptors for different CrRLK1L proteins in diverse developmental contexts.

### Downstream Signaling Pathways

Downstream components of CrRLK1L signaling have been identified, including RAC/ROP guanine exchange factors (ROPGEFs), a plant-specific subfamily of RHO-GTPases that convert GDP into GTP and activate RAC/ROPs (Carol et al., 2005). Two studies have identified five ROPGEFs that interact with the kinase domain of FER, and two RAC/ROPs as potential downstream components (Duan et al., 2010; Yu et al., 2012). One RAC/ROP (ROP11; AT5G62880) phosphorylates and inactivates PP2C phosphatase ABI2, an integral element in abscisic acid (ABA) signaling (Nishimura et al., 2010; Yu et al., 2012). Adding further complexity, the stunted phenotype of *fer-4* mutants has been linked to the ethylene biosynthetic pathway through characterization of *S*-adenosylmethionine synthetase 1 and 2 (SAM1 and SAM2) as direct interactors of the FER kinase domain (Mao et al., 2015). Mao et al. (2015) propose a scenario in which FER inhibits SAM1 and SAM2 through phosphorylation, suppressing *S*-adenosylmethionine production and down-regulating ethylene biosynthesis. Together, these findings depict the first steps in a branched CrRLK1L pathway, where a single receptor can interact with multiple partners to activate different downstream elements (**Figure 1B**). Crosstalk of CrRLK1L signaling with the hormones ABA, ethylene, and BR is also likely.

As previously discussed, increased ROS production occurs in various CrRLK1L-mediated processes. NADPH oxidases are required for the CrRLK1L-mediated ROS burst during tip growth in root hairs or PTs, and PT reception at the SCs (Lee and Yang, 2008; Boisson-Dernier et al., 2013). Interestingly, studies in rice identified a NADPH oxidase as a direct interactor of a RAC/ROP protein (Wong et al., 2007). This connection has recently been confirmed in *Arabidopsis* by characterizing ROP11 as an interaction partner and activator of the NADPH oxidase Rboh F in root hairs (Yan et al., 2016). Finally, NTA and the receptor-like cytoplasmic kinase MARIS also act downstream in this signaling cascade (Kessler et al., 2010; Boisson-Dernier et al., 2015), although the exact mechanisms are yet to be elucidated. The question of whether CrRLK1L pathways indirectly modify gene expression by post-translational modification of transcription factors remains to be addressed.

## An Evolutionary Perspective of the CrRLK1L Subfamily

Despite recent advances, only four studies focus on CrRLK1L protein function in species other than *Arabidopsis*. Beyond the initial identification of CrRLK1 (Schulze-Muth et al., 1996), phylogenomics identified 16 CrRLK1L homologs with Gigantea-mediated circadian regulation of their expression in *Oryza sativa* (Nguyen et al., 2014). Secondly, Niu et al. (2016) described ∼40 CrRLK1L family members in diploid cotton species, six of which are linked to fiber development. Finally, a closely related protein, CpRLK1, was functionally characterized in a charophycean unicellular alga (Hirano et al., 2015). CpRLK1 is expressed during mating in this heterothallic alga and regulates gamete formation. Hirano’s study is of particular evolutionary interest since comparison of CrRLK1L function between algae and angiosperms demonstrates potentially analogous involvement in reproduction, cell growth, and cell wall stability sensing. CpRLK1 functions in the release of +-type gametes that lack a cell wall, with +-type cells of CpRLK1 knockdown lines generating an abnormally enlarged conjugation papilla but not releasing the gametal protoplast (Hirano et al., 2015). CpRLK1 is suggested to detect cell wall integrity during conjugation to regulate the release of +-type cells, evoking similarities with regulation of PT function by CrRLK1L proteins in angiosperms (Wolf and Hofte, 2014). Similarly, partial protoplast protrusion in *Closterium* CpRLK1 knockdown lines resembles cellular material discharge in impaired root hair development phenotypes of *fer-4* mutants, supporting a possible ancestral role in cell wall integrity surveillance (Li et al., 2015).

Plant RLKs may have originated in the Streptophyta lineage, which is ancestral to both land plants and charophycean algae (Sasaki et al., 2007). Although, limited by a paucity of algal genomes or transcriptomes, CpRLK1 identification in a charophycean alga substantiates divergence of RLK subfamilies and emergence of the CrRLK1L subfamily early in the evolution of Streptophytes (Shiu and Bleecker, 2001; Sasaki et al., 2007). Conservation of cell wall synthesizing enzymes (rosette cellulose, arabinogalactan, and hemicellulose synthases) across the Streptophyta lineage supports the hypothesis of evolutionary conservation of CrRLK1L cell wall surveillance functions (Sorensen et al., 2010; Popper et al., 2011; Fangel et al., 2012).

**FIGURE 2:**
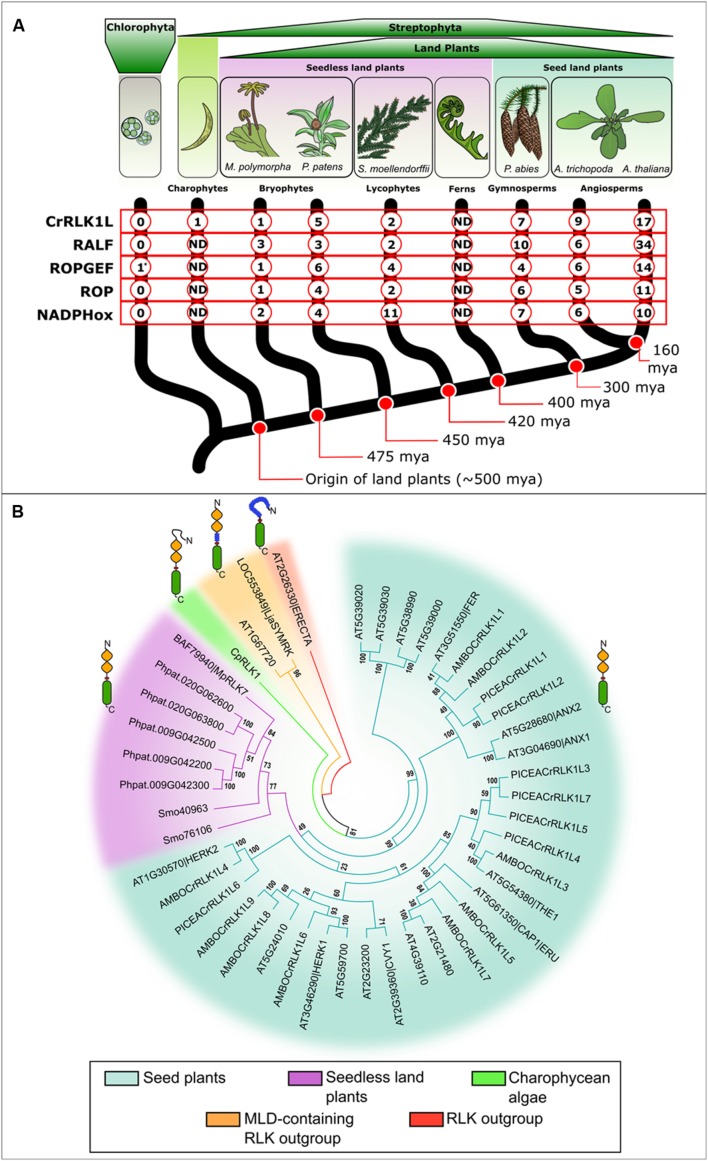
**(A)** Occurrence of CrRLK1L signaling elements across plant lineages. Data is shown for Chlorophyta species *Ostreococcus lucimarinus*, *Chlamydomonas reinhardtii*, *Volvox carteri*, *Coccomyxa subellipsoidea*, *Micromonas pusilla*, and *Micromonas* sp. (Merchant et al., 2007; Palenik et al., 2007; Worden et al., 2009; Prochnik et al., 2010; Blanc et al., 2012); Charophyta *Closterium peracerosum-strigosum-littorale* complex (Hirano et al., 2015); the liverwort *Marchantia polymorpha* (Sasaki et al., 2007; These sequence data were produced by the US Department of Energy Joint Genome Institute http://www.jgi.doe.gov/ in collaboration with the user community); the model bryophyte *Physcomitrella patens* (Rensing et al., 2008; Lehti-Shiu and Shiu, 2012); lycophyte *Selaginella moellendorffii* (Banks et al., 2011; Lehti-Shiu and Shiu, 2012); conifer *Picea abies* (Nystedt et al., 2013); single living sister species to all other angiosperms *Amborella trichopoda* (Albert et al., 2013) and *Arabidopsis thaliana* (Hematy and Hofte, 2008). According to the GenBank, Phytozome and ConGenIE databases, the CrRLK1L subfamily is only present in Streptophyta, increasing in number with developmental complexity. Other signaling elements present similar trends in numbers: RALF peptides, ROPs and membrane bound NADPH oxidases are absent in Chlorophyta and present in land plants (Berken et al., 2005; Torres et al., 2006; Eklund et al., 2010; Cao and Shi, 2012). ROPGEFs are present in land plants and Chlorophyta, although their presence in the latter is restricted to *Ostreococcus lucimarinus* (^∗^). Full genomic or transcriptomic records are not available for charophyte and fern lineages included in this figure (ND). **(B)** Comparative phylogenetic analysis of the *C*rRLK1L proteins in plants. Sequences were aligned using ClustalX (Larkin et al., 2007). The evolutionary history was inferred using a Neighbor-Joining phylogenetic tree generated with the software MEGA5.2 (Saitou and Nei, 1987; Tamura et al., 2011). The percentage of replicate trees in which the associated taxa clustered together in the bootstrap test (1000 replicates) is shown next to each branch. Putative CrRLK1L members in *A. thaliana* (AT4G39110, AT2G21480, AT5G61350|CAP1|ERU, AT5G54380|THE1, AT2G23200, AT5G24010, AT2G39360|CVY1, AT3g46290|HERK1, AT5g59700, AT1g30570|HERK2, AT3G51550|FER, AT3G04690|ANX1, AT5G28680|ANX2, AT5G39000, AT5G38990, AT5G39020, AT5G39030), *A. trichopoda* (AMBOCrRLK1L1-9; Phyotzome v11 IDs: evm_27.TU.AmTr_v1.0_scaffold – 00077.136, 00016.362, 00003.355, 00092.136, 00045.60, 00080.56, 00024.19, 00001.334, 00001.335), *P. abies* (PICEACrRLK1L1-7; ConGenIE IDs: MA_71280g0010, MA_21765g0010, MA_10432359g0010, MA_5246g0010, MA_44655g0010, MA_45223g0010, MA_10432359g0020), *S. moellendorffii* (Smo40963, Smo76106), *P. patens* (Phpat.020G063800, Phpat.020G062600, Phpat.009G042500, Phpat.009G042200, Phpat.009G042300), *M. polymorpha* (BAF79940|MpRLK7), and *Closterium peracerosum-strigosum-littorale* Complex (AB920609|CpRLK1) are shown. *Lotus japonicus* MLD-LRR-containing RLK SYMRK (LOC553849|LjaSYMRK) and its closest MLD-LRR-RLK homolog in *A. thaliana* (AT1G67720) were used as an outgroup together with ERECTA from *A. thaliana* (AT2G26330|ERECTA).

There has been an expansion of the CrRLK1L subfamily from early diverging to recent Streptophyta lineages, with the greatest increase in prevalence evident between seedless vascular plants and seed plants (**Figure 2A**). Expansion of the subfamily may have allowed acquisition of new roles in reproductive processes such as PT growth and its recognition at the female gametophyte (see Pollen Tube-Female Gametophyte Interactions), developmental innovations characteristic of the seed plants (Doyle, 2006; Linkies et al., 2010). An extant member of the earliest divergent angiosperm lineage, *Amborella trichopoda* (Albert et al., 2013) has orthologs of most characterized *A. thaliana* CrRLK1L proteins (**Figure 2B**). The conifer *Picea abies* (Nystedt et al., 2013) has orthologs of the reproductive clade (FER, ANX1, and ANX2) and some non-reproductive members (THE1 and HERK2; **Figure 2B**). FER and ANX orthologs in *A. trichopoda* and *P. abies* suggest a potentially conserved role of these CrRLK1Ls in mediating PT growth and fertilization (see Pollen Tube-Female Gametophyte Interactions). Fertilization in ferns and some gymnosperms (e.g., cycads and ginkgo) depends on multiflagellated sperm rather than on siphonogamous PT transport of non-motile sperm cells, which arose independently in angiosperms and gymnosperms (Doyle, 2006). Given available data, it is impossible to speculate whether the reproductive clade formed independently when siphonogamy established in conifers and angiosperms or was already present before divergence of these lineages of land plants.

Although *Selaginella moellendorffii* is a vascular plant, the composition of its CrRLK1L family closely resembles that of non-vascular plants: CrRLK1L subfamily members from the liverwort *Marchantia polymorpha*, the moss *Physcomitrella patens*, and the lycophyte *S. moellendorffii* form a sister clade to non-reproductive CrRLK1L proteins (THE1, CVY1, HERK1, and CAP1; see Cell Wall Integrity, Cell Elongation, Tip Growth in Root Hairs, Pollen Tubes, and Trichomes). Although further research is required to confirm protein function, the phylogeny suggests a potentially conserved function in cell wall stability and regulation of cell elongation across land plants that was recruited in seed plant specific processes as the family expanded. Algal CpRLK1 does not cluster with the early divergent Streptophyta sequences, but is positioned between the CrRLK1L subfamily and the closely related MLD-LRR-RLK subfamily, of which *Lotus japonicus* SYMRK is best characterized (Antolin-Llovera et al., 2014; Hirano et al., 2015). This may be due to additional extracellular sequences in both CpRLK1 and MLD-LRR-RLKs, which are not present in other CrRLK1L proteins. Ongoing genomic and transcriptomic projects will facilitate more comprehensive phylogenetic studies of RLKs in early diverging Streptophyte lineages and ferns. Phylogenies along with functional studies from diverse species will help us decipher (i) the importance of divergence between CpRLK1 and other CrRLK1Ls, (ii) the evolutionary history of the expansion of this subfamily of RLKs, and (iii) whether the reproductive clade of CrRLK1Ls is specific to seed plants or appeared earlier in the evolution of plants, i.e. in ferns.

Interestingly, CrRLK1L signaling may be conserved from bryophytes to angiosperms (**Figure 2A**). Downstream signaling components for polarized cell expansion and tip growth mechanisms that are CrRLK1L-mediated are present in liverworts, bryophytes, lycophytes, gymnosperms and angiosperms, including ROPGEFs, RAC/ROPs and NADPH oxidases. Homologs of putative ligands in the form of RALF peptides are also present in these species (Cao and Shi, 2012; Albert et al., 2013; Nystedt et al., 2013). Furthermore, ROPGEFs and RAC/ROPs have conserved functions in cell polarity and tip growth regulation in the filamentous gametophytic (protonemal) stage of *P. patens* (Ito et al., 2014). In this light, it is plausible that CrRLK1L proteins mediate tip growth from bryophytes to angiosperms via conserved CrRLK1L-ROPGEF-RAC/ROP signaling pathways. A more detailed analysis of when signaling pathway components arose relative to CrRLK1L kinases is precluded by a lack of genomic data from early diverging Streptophyta lineages (excepting the identification of ROPGEF proteins in some Chlorophyta species), however, all signaling components were apparently present in early non-vascular land plants (**Figure 2A**).

As a final example of how CrRLK1L function and signaling may be conserved across plant evolution, let us consider CAP1 function in root development. Rhizoids are tip-growing root-like structures providing anchorage and water and nutrient uptake for some charophytes and land plants with a free-living gametophytic generation (liverworts, mosses, hornworts, lycophytes, and ferns; Jones and Dolan, 2012). Rhizoids and root hair development share a regulatory transcription network in which transcription factor ROOTHAIR DEFECTIVE 6 (RHD6) family has a central function. RHD6 transcription factors drive expression of root hair-specific genes, including genes required for cell expansion, and are necessary and sufficient for root hair and rhizoid development in angiosperms, liverworts and mosses (Masucci and Schiefelbein, 1994; Menand et al., 2007; Yi et al., 2010). Interestingly, expression of CAP1 (see Tip Growth in Root Hairs, Pollen Tubes, and Trichomes) is positively regulated by RHD6 and RHD6-LIKE 4 (RSL4) in *Arabidopsis*, suggesting that CAP1 acts downstream of RHD6 and RSL4 in root hair growth (Menand et al., 2007; Bruex et al., 2012). Given functional conservation of RHD6 across distant land plant lineages, we can hypothesize that a similar regulatory network may occur in early diverging land plants, in which RHD6/RSL4 orthologs transcriptionally regulate CrRLK1Ls during tip growth of rhizoids.

## Future Directions

CrRLK1L kinases are receiving increasing attention for their roles in regulating developmental and stress responses. A number of questions remain to be addressed: (i) cross-talk with hormonal pathways, (ii) putative carbohydrate-binding capacity via the MLD, and (iii) the ligand-receptor relationships with different RALF peptides. Functional conservation across plant lineages remains speculative, with further research in early diverging lineages key to inferring primordial function, and understanding conserved mechanisms shared by functionally diverse CrRLK1L proteins in higher plants.

## Author Contributions

SG-T, JG, and LS conceived and designed the review. SG-T wrote the manuscript draft and all authors edited, read and approved the final manuscript.

## Conflict of Interest Statement

The authors declare that the research was conducted in the absence of any commercial or financial relationships that could be construed as a potential conflict of interest.

## ABBREVIATIONS

ABA: abscisic acid
ANX1: ANXUR1
ANX2: ANXUR2
BR: brassinosteroid
CAP1/ERU: [Ca^2+^]_cyt_-associated protein kinase 1/ERULUS
CpRLK1: *Closterium peracerosum-strigosum-littorale* Complex RLK 1
CrRLK1L: *Catharanthus roseus* RLK 1-like
CVY1: CURVY1
ECD: extracellular domain
FER: FERONIA
flg22: flagellin 22 (epitope of FLAGELLIN)
GPI-AP: glycosylphosphatidylinositol-anchored protein
HERK1: HERCULES Receptor Kinase 1
HERK2: HERCULES Receptor Kinase 2
LLG: LRE-like GPI-AP
LRE: LORELEI
LRR: leucine-rich repeat
MD: malectin domain
MLD: malectin-like domain
MLO: mildew resistance locus O
NTA: NORTIA
PAMP: pathogen-associated molecular pattern
PM: powdery mildew
PT: pollen tube
RALF: rapid alkalinization factor
RLK: receptor-like kinase
ROPGEF: RAC/ROP guanine exchange factor
ROS: reactive oxygen species
SAM1: *S*-adenosylmethionine synthetase 1
SAM2: *S*-adenosylmethionine synthetase 2
SC: synergid cells
THE1: THESEUS1
TM: transmembrane domain.
